# The complete mitochondrial genome of *Enallagma cyathigerum* (Odonata: Coenagrionidae) and phylogenetic analysis

**DOI:** 10.1080/23802359.2017.1375879

**Published:** 2017-09-09

**Authors:** Li Zhang, Xiao-Tong Wang, Chun-Li Wen, Meng-Yao Wang, Xing-Zhuo Yang, Ming-Long Yuan

**Affiliations:** State Key Laboratory of Grassland Agro-Ecosystems, College of Pastoral Agricultural Science and Technology, Lanzhou University, Lanzhou, Gansu, People's Republic of China

**Keywords:** Odonata, Zygoptera, damselflies, mitochondrial DNA, phylogeny

## Abstract

To better understand the diversity and evolution of Odonata, we sequenced and annotated the complete mitochondrial genome (mitogenome) of *Enallagma cyathigerum*. This mitogenome was 16,661 bp in size and encoded the typical 37 genes, i.e. 13 protein-coding genes (PCGs), 22 transfer RNA genes (tRNAs) and two ribosomal RNA genes. The nucleotide composition of the *E*. *cyathigerum* mitogenome was significantly biased toward A and T, with an A + T content of 74.2%. Eleven PCGs started with a typical ATN codon, whereas the remaining two PCGs (*nad1* and *nad3*) used TTG as the initial codon. All the 22 tRNAs had a typical secondary cloverleaf structure. The Bayesian phylogenetic analysis based on the concatenated nucleotide sequences of 13 PCGs strongly supported the sister relationship of *E*. *cyathigerum* and two *Ischnura* species from the same family Coenagrionidae. The phylogenetic tree strongly supported the monophyly of each of the two suborders (Zygoptera and Anisoptera) and recovered a phylogeny of Zygoptera + (Anisoptera + Anisozygoptera).

## Introduction

The Odonata is an excellent model to study insect ecology and evolution, and comprised of three suborders (Zygoptera, Anisoptera, and Anisozygoptera) with approximately 6000 described species (Zhang 2013; Bybee et al. 2016). Although considerable research effort has been expended on Odonata (Dumont et al. 2010; Carle et al. 2015; Feindt et al. 2016; Yong et al. 2016), phylogenetic relationships within Odonata are still unresolved. In this study, we sequenced and annotated the complete mitogenome of *Enallagma cyathigerum* (Odonata: Coenagrionidae). This is the third completely sequenced mitogenome from the family Coenagrionidae. The adult specimens of *E*. *cyathigerum* were collected from Altay Prefecture, Xinjiang, China, in August 2016. Samples have been deposited in College of Pastoral Agricultural Science and Technology, Lanzhou University, Lanzhou, China. The total genomic DNA was extracted from the head of a single specimen using a DNeasy Tissue Kit (Qiagen, Valencia, CA) according to the protocols from the manufacturer. The entire mitogenome of *E*. *cyathigerum* was amplified with a set of universal and specific primer pairs, and sequenced in both directions.

The complete mitogenome of *E*. *cyathigerum* was a typical circular DNA molecule with 16,661 bp in length (GenBank accession number MF716899). This mitogenome is the second largest among all sequenced mitogenomes of Odonata, primarily due to the significant size increase of the putative control region (1906 bp). The *E*. *cyathigerum* mitogenome encoded a typical set of 37 mitochondrial genes, i.e. 13 protein-coding genes (PCGs), 22 transfer RNA genes (tRNAs) and two ribosomal RNA genes (*rrnL* and *rrnS*). The arrangement and orientation of the mitochondrial genes are identical to that of the putative ancestral insect mitogenome. Gene overlaps were found at eight gene junctions and involved a total of 35 bp, with the longest overlap (8 bp) between *trnW* and *trnC*, whereas a total of 65 bp intergenic spacers were present in 10 positions, ranging in size from 1 to 17 bp.

The *E*. *cyathigerum* mitogenome with an A + T content of 74.2% showed a positive AT-skew (0.107) and a negative GC-skew (–0.096) on the J-strand. Among the 13 PCGs, the lowest A + T content was 67.3% in *cox3*, while the highest was 80.9% in *atp8*. All the 22 tRNAs, ranging from 65 bp to 72 bp, had a typical cloverleaf secondary structure. The *rrnL* was 1286 bp long with an A + T content of 75.9%, while the *rrnS* was 760 bp long with an A + T content of 74.5%.

**Figure 1.**
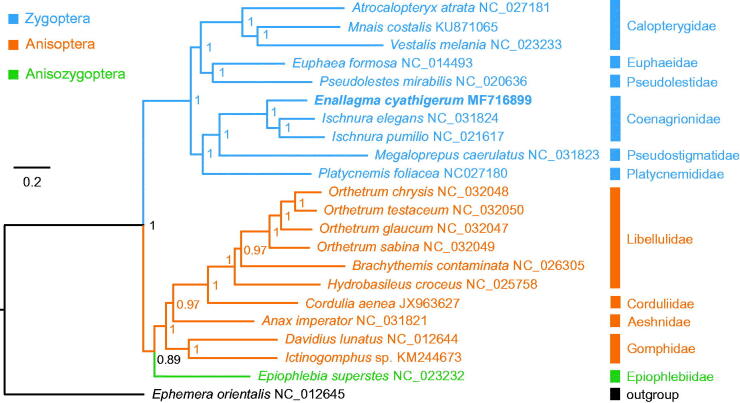
Mitochondrial phylogeny of 21 Odonata species based on the concatenated nucleotide sequences of 13 mitochondrial protein-coding genes.

Based on the concatenated nucleotide sequences of 13 PCGs from 21 Odonata species and an outgroup from the Ephemeroptera (*Ephemera orientalis*), we conducted a Bayesian phylogenetic analysis following the methods described in Tang et al. (2017). The monophyly of each of the two suborders (Zygoptera and Anisoptera) was strongly supported and a phylogeny of Zygoptera + (Anisoptera + Anisozygoptera) was recovered (Figure 1). The phylogenetic analysis strongly supported the sister relationship of *E*. *cyathigerum* and two *Ischnura* species from the same family Coenagrionidae (posterior probability =1.0). The family relationships in the Zygoptera were (Calopterygidae + (Euphaeidae + Pseudolestidae)) + (Platycnemididae + (Coenagrionidae + Pseudostigmatidae)). For the Anisoptera, a phylogeny of Gomphidae + (Aeshnidae + (Libellulidae + Corduliidae)) was strongly supported.
